# Electron-Beam-Lithographed Nanostructures as Reference Materials for Label-Free Scattered-Light Biosensing of Single Filoviruses

**DOI:** 10.3390/s18061670

**Published:** 2018-05-23

**Authors:** Anant Agrawal, Joseph Majdi, Kathleen A. Clouse, Tzanko Stantchev

**Affiliations:** 1 Division of Biomedical Physics, Office of Science and Engineering Laboratories, Center for Devices and Radiological Health, Food and Drug Administration, Silver Spring, MD 20993, USA; joemajdi@yahoo.com; 2 Division of Biotechnology Review and Research I, Office of Biotechnology Products, Office of Pharmaceutical Quality, Center for Drug Evaluation and Research, Food and Drug Administration, Silver Spring, MD 20993, USA; kathleen.clouse@fda.hhs.gov (K.A.C.); tzanko.stantchev@fda.hhs.gov (T.S.)

**Keywords:** optical biosensing, electron beam lithography, label-free, filovirus, reference materials, scattered light, interferometry

## Abstract

Optical biosensors based on scattered-light measurements are being developed for rapid and label-free detection of single virions captured from body fluids. Highly controlled, stable, and non-biohazardous reference materials producing virus-like signals are valuable tools to calibrate, evaluate, and refine the performance of these new optical biosensing methods. To date, spherical polymer nanoparticles have been the only non-biological reference materials employed with scattered-light biosensing techniques. However, pathogens like filoviruses, including the Ebola virus, are far from spherical and their shape strongly affects scattered-light signals. Using electron beam lithography, we fabricated nanostructures resembling individual filamentous virions attached to a biosensing substrate (silicon wafer overlaid with silicon oxide film) and characterized their dimensions with scanning electron and atomic force microscopes. To assess the relevance of these nanostructures, we compared their signals across the visible spectrum to signals recorded from Ebola virus-like particles which exhibit characteristic filamentous morphology. We demonstrate the highly stable nature of our nanostructures and use them to obtain new insights into the relationship between virion dimensions and scattered-light signal.

## 1. Introduction

Label-free optical biosensors are being developed to fill the need for rapid, inexpensive, and field-deployable detection of specific pathogenic microorganisms like Ebola virus (EBOV), the cause of one of the most severe outbreaks of deadly hemorrhagic fever during the years 2014–2016. In principle, these biosensors accept a small sample of body fluid and the target pathogens present in the sample are directly captured, optically interrogated, and identified with minimal operator interaction. One broad category of optical biosensors uses scattered light to detect and characterize single virions [1,2,3,4,5,6,7,8,9,10], which as dielectric nanoparticles yield very weak scattering, but the tiny optical field is measurable and information-rich. This “scattered-light signal” (from here, referred to simply as “signal”) is strongly influenced by the three primary physical properties of a dielectric nanoparticle—shape, size, and refractive index—all of which facilitate agile pathogen identification.

At various stages of the life cycle of a biosensor—from design to deployment—reference materials are needed to compare prototype sensor configurations, calibrate sensor parameters, verify sensor quality at manufacture, and ensure consistent sensor performance over time in the field. For single-nanoparticle optical biosensing strategies, spherical polymer nanoparticles (i.e., polystyrene or silica) have been the biohazard-free reference material of choice to calibrate and evaluate biosensor performance [1,3,6,7,10]. These nanoparticles roughly match the refractive index of the target microorganism and are commercially available in the relevant sizes. However, when the target is non-spherical in nature, as is the case with filoviruses, reference materials with similar nanoscale morphology can greatly benefit biosensor calibration, evaluation, and refinement.

In this work, we have fabricated and characterized a new type of reference material realized as nanostructures closely resembling the morphology and refractive index of filoviruses, namely EBOV. Fabrication was achieved with electron beam lithography (EBL) of hydrogen silsesquioxane (HSQ) on a substrate specific to a promising scattered-light biosensing scheme based on interferometric reflectance imaging [1,2,3,4], though the nanostructures are readily fabricated on other substrates as well. Upon fabrication, we measured the nanostructures’ dimensions with scanning electron and atomic force microscopes for subsequent correlation to signal. We assessed the similarity of the nanostructures’ signals to those from EBOV virus-like particles (VLPs), which have morphology resembling that of the wild-type EBOV [11,12]. We observed notable consistency of the signals over a 9-month period (coefficient of variation (CV) < 2%) from a collection of nanostructures on a chip stored simply in a Petri dish on a lab bench. Finally, we used the nanostructures to reveal the polarization-dependent signal from asymmetric objects, a characteristic which can enhance detection of EBOV virions and other asymmetric microorganisms.

## 2. Materials and Methods

### 2.1. Key Virion Characteristics: Shape, Size, and Refractive Index

Filoviruses such as EBOV are long and tubular in shape with length ranging from hundreds to thousands of nanometers. The shortest virions generally appear as straight line segments, occasionally with a bulbous end, while longer virions more likely have curves. Recent electron microscopy studies have reported 96–99 nm diameter for EBOV virions either frozen-hydrated [13] or treated with ionic liquid [14]. The EBOV genome size of 18.9 kilobases suggests a theoretical virion length of 980 nm, which is consistent with cryo-electron microscopy findings that most virus particles have a length of 982 ± 79 nm, though significantly longer polyploid virions were also observed [13]. We assume a filovirus refractive index of 1.42, based on a recent report of the first-ever experimental refractive index measurement of individual animal virions (HIV-1) [15].

### 2.2. Electron Beam Lithography (EBL)

EBL is a direct-write process to create structures with nanoscale detail by scanning a focused electron beam in a precise pattern over a surface coated with a thin film of electron-sensitive material (resist) [16]. Immersion of the film in the appropriate developer solution after electron beam exposure leads to removal of either the exposed or unexposed resist from the surface, depending on whether the resist is positive-tone or negative-tone, respectively. The EBL system we used in this work comprised the Nanometer Pattern Generation System (JC Nabity Lithography Systems, Bozeman, MT, USA) coupled to a scanning electron microscope (SEM, JSM-6400, JEOL Ltd., Tokyo, Japan). This system accepts computer-aided design (CAD) drawings of the patterns to be traced with the electron beam on the resist. We drew patterns of individual virions represented as simple rectangles of various lengths and widths, with large separation between the rectangles to easily isolate the signal from each.

**Figure 1:**
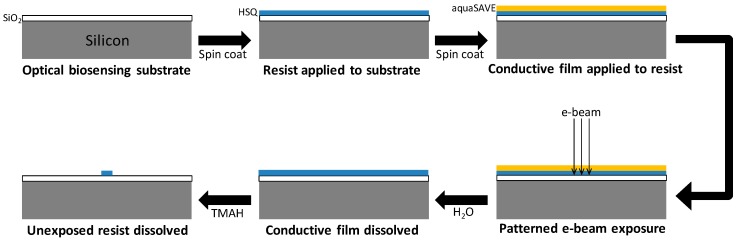
Electron beam lithography process. Dimensions are not to scale.

We chose HSQ as the resist with which to fabricate our nanostructures, in part because HSQ is negative-tone and therefore more efficient than positive-tone for fabricating the spread-out collection of nanostructures we desired. Furthermore, from reported ellipsometry measurements of HSQ films [17] and our own Abbe refractometer measurements of the liquid resist, HSQ refractive index is 1.39 ± 0.01, closely matching the assumed virion index of 1.42. Our specific EBL process is illustrated in Figure 1. We spin coated HSQ (XR-1541-002 or -004, Dow Corning Corp., Midland, MI, USA) onto the biosensing substrate (described in the next section), followed by soft bake on a hot plate at 150 °C for 5 min to evaporate solvent. To prevent troublesome charge accumulation during EBL, we spin coated a water-soluble conductive film (aquaSAVE, Mitsubishi Chemical Corp., Tokyo, Japan) on top of the HSQ film, followed by soft bake at 100 °C for 2 min to evaporate solvent. The CAD patterns were then written onto the chip with the following system parameters, which we settled upon after a number of trials: 30 kV acceleration voltage, 50 µm aperture, 16 mm working distance, 4625 µC/cm^2^ energy dose. After electron beam exposure, the chip was briefly immersed in distilled water to remove the aquaSAVE film and then dried with a purified N_2_ stream. Finally, the chip was immersed in a developer solution of 25% tetramethylammonium hydroxide (TMAH) in water (331635, Sigma-Aldrich Inc., St. Louis, MO, USA) for 23 s to remove unexposed resist, rinsed with distilled water and dried with N_2_.

We imaged the fabricated nanostructures on the chip with field emission SEM (SU-70, Hitachi High Technologies America, Inc., Schaumburg, IL, USA) to measure their length and width, and we used an atomic force microscope (AFM, MFP-3D, Asylum Research Inc., Santa Barbara, CA, USA) to measure thickness. SEM images were analyzed with ImageJ [18] and MATLAB (Mathworks, Natick, MA, USA), while AFM images were analyzed with Igor Pro (Wavemetrics, Lake Oswego, OR, USA) integrated into that microscope’s software interface.

### 2.3. Scattered-Light Biosensing with Interferometric Reflectance Imaging

**Figure 2:**
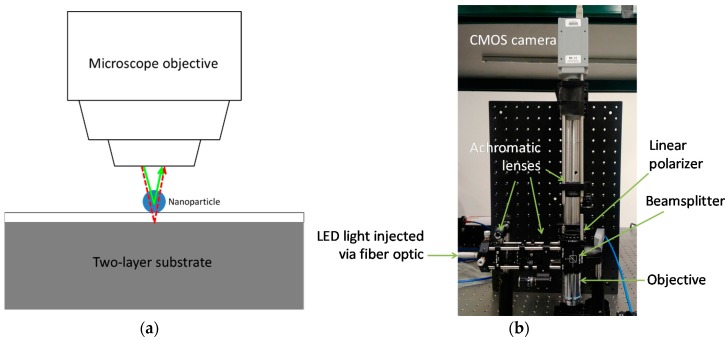
Interferometric reflectance imaging for biosensing. (**a**) Illustration of the basic principle. The solid green line approximates a sample path of light waves reflected off the nanoparticle, and the dashed red line approximates a reference path reflection from the interface between the two substrate layers. Dimensions are not to scale. (**b**) Photograph of our laboratory biosensor setup.

Interferometric sensing enables detection of astoundingly small optical fields, by mixing the small field with a much larger reference field. When the weakly reflected light from a spherical nanoparticle interferes with reference light from a strong reflector, the detected optical intensity (*I*) relates to particle radius (*r*) as *I* ∝ *r*^3^, which is a dramatically increased sensitivity to the smallest particles over direct detection of a nanoparticle’s reflected light, for which the relationship is *I* ∝ *r*^6^ [1,19]. As illustrated in Figure 2a, this sensing arrangement can be realized as a common-path interferometer by placing a nanoparticle on top of a two-layer substrate, with top layer thickness similar to the particle size and the bottom layer effectively semi-infinite, and illuminating from above. Light reflecting from the particle measurably interferes with the reflected light from the underlying interface between the substrate’s top and bottom layers serving as the reference. In addition to dependence on particle size, the nature (constructive or destructive) and magnitude of the interference depends on the particle’s refractive index, the illumination wavelength, and the thickness of the substrate’s top layer.

Based on the groundbreaking work from the Ünlü research group [1,2,3,4], we implemented an interferometric reflectance imaging biosensor as an epi-illumination brightfield microscope (Figure 2b). A multi-LED light source (WLS-22-A, Mightex Systems, Pleasanton, CA, USA) is fiber-optically coupled to an achromatic relay lens pair whose focal plane is aligned with the back focal plane of a 50×/0.8 NA infinity-corrected microscope objective (LU Plan EPI, Nikon, Tokyo, Japan), thus providing Köhler illumination onto the biosensing substrate. This objective is designed to be used in air without a coverslip at 1 mm working distance. A 50/50 cube beamsplitter reflects light from the relay lenses to the objective, and light reflecting from the sample returns through the objective and passes through the beamsplitter, to an achromatic tube lens which images onto a monochrome CMOS camera (PL-B771U, Pixelink, Ottawa, ON, Canada). A rotatable linear polarizer was inserted between the beamsplitter and tube lens for some of our imaging, allowing for isolation of any linear polarization state at the camera. The two-layer substrate is a silicon wafer coated with a 100 nm-thick thermal silicon oxide (SiO_2_) film (Silicon Valley Microelectronics, Santa Clara, CA, USA).

With target objects on the substrate in focus within the microscope’s field of view, we captured images sequentially with eight different narrowband LEDs whose nominal central wavelengths are 400, 455, 470, 505, 530, 590, 625, 656 nm. We also captured background images (no LED illumination) to account for ambient light and camera digital offset contributions to the images at each of the LED wavelengths. We then used routines written in MATLAB with the acquired images to generate an 8-point spectrum (signal versus LED wavelength) for each object on the substrate.

### 2.4. EBOV Virus-Like Particles (VLPs)

**Figure 3:**
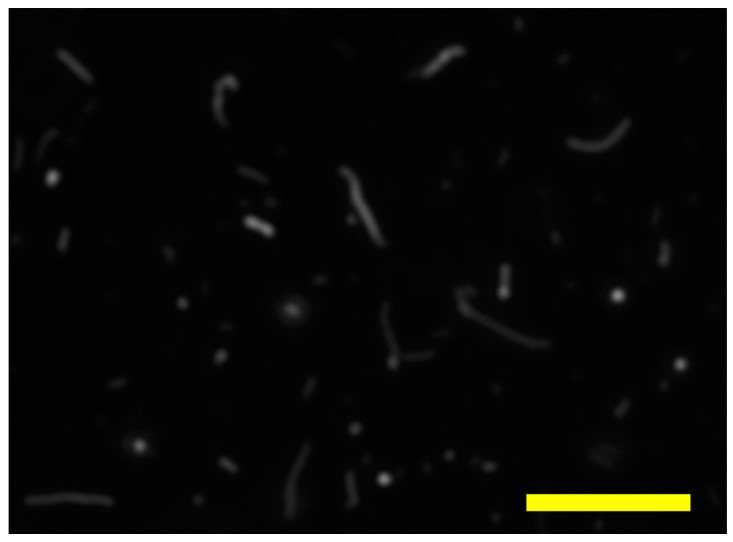
Fluorescence microscopy of EBOV VLPs, demonstrating filamentous morphology with varying lengths. Scale bar is 5 µm.

Cell fusion-capable but replication-incompetent EBOV surface glycoprotein (GP)-pseudotyped VLPs have emerged as a viable alternative to study filoviruses under biosafety level 2 (BSL-2) conditions [20,21]. The VLPs used in the current study were produced by co-transfecting 293T cells with a plasmid encoding the EBOV GP strain Kikwit (VRC 6001), a plasmid encoding EBOV matrix protein VP40 (pCAGG VP40), and a plasmid encoding VP40 linked to green fluorescent protein (VP40-GFP). 12–16 h after co-transfection the 293T cells were gently washed and incubated in fresh Dulbecco’s Modified Eagle’s Medium supplemented with 10% fetal calf serum, l-glutamine and non-essential amino acids. The VLP-containing cell culture medium was collected after 24 and 48 h, cleared from cell debris by low speed centrifugation and passed through a 0.45 µm low protein binding filter. The VLPs were further concentrated by ultracentrifugation through a 20% sucrose cushion as previously described [12]. We then removed the sucrose from the VLP suspension via microdialysis with an aqueous 0.5% NaCl solution as the dialysate. It was previously demonstrated that VLPs generated using VP40 display the characteristic filamentous morphology [11,12], and we confirmed this morphology via fluorescence microscopy with a blue excitation/green emission filter set to visualize GFP (Figure 3).

For biosensing experiments, a 0.5 µL droplet of the dialyzed VLP suspension was pipetted onto a clean biosensing substrate and placed into a humidity chamber for 1 h to allow VLPs to settle. Upon removal from the humidity chamber, the excess moisture of the droplet was carefully absorbed with a laboratory wipe. We identified isolated VLPs bound to the substrate surface with fluorescence microscopy prior to biosensing measurements.

## 3. Results

### 3.1. Nanostructure Dimensions

**Figure 4:**
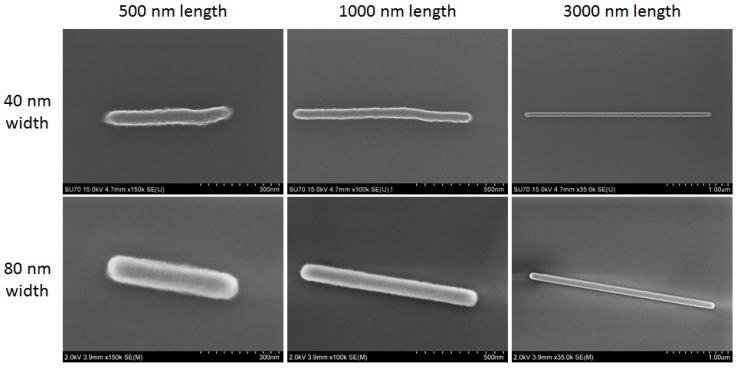
Scanning electron microscope (SEM) images of six fabricated nanostructures. Labeled lengths and widths are design values. Scale bars vary for each image as shown.

We fabricated a total of 48 nanostructures, comprised of eight groups of six different lengths. The eight groups arise from having duplicate sets of two different widths and two different orientations (vertical and horizontal). Thickness was defined primarily by the HSQ resist spin coat process and electron beam dose [22]. Figure 4 shows representative SEM images of the nanostructures. Though designed as rectangles, the final shapes have rounded ends due to the expected blurring effect of the electron beam’s point spread function. Rough edges in all structures result from randomness in electron and developer interactions with the resist. More gross shape errors were apparent in some structures (e.g., 500 × 40 nm and 1000 × 40 nm structures shown in Figure 4) due to occasional irregularities in the EBL system operation. All of these deviations from the ideal rectangular shape were considered negligible given their size relative to the overall structure size and to the wavelengths of light for biosensing.

Table 1 summarizes the dimensions of all the fabricated nanostructures. Measured values represent the mean ± one standard deviation, with the CV shown in parentheses. The SEM image of one of the 2000 nm-long structures was corrupted, so we took the mean length from only three structures instead of four. Overall, we achieved excellent consistency in length, with CV generally shrinking as structure length increased; width and thickness were slightly less consistent. Measured length was always slightly larger than design length, while measured width was considerably larger than designed. We attribute these results primarily to the same blurring effect which rounded the ends of the structures. Uncertainty in the spin coat process and electron beam-HSQ interaction gave rise to the thickness error, which we consider to be reasonably small.

### 3.2. Unpolarized Signals from Nanostructures

**Figure 5:**
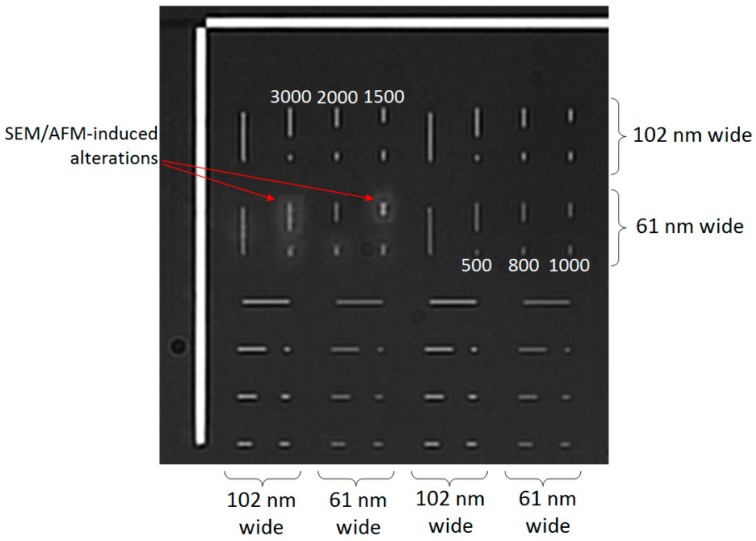
Scattered-light image of nanostructures on biosensing substrate with 530 nm illumination. The six different design lengths (in nm) are labeled in white across two of the eight groups.

Figure 5 shows a representative biosensor image of our 48 nanostructures with 530 nm illumination. Additional structures with other lengths and widths were included in the EBL pattern to assist with orientation and positioning. Since SEM or AFM could alter a nanostructure and the adjacent surface, as is evident from the brightness variations of the left group of vertical 61 nm-wide nanostructures, one set of 24 was used for SEM/AFM measurements and the other set was left untouched for optical imaging. For our biosensor setup with NA of 0.8 and illumination wavelengths ranging from 400 to 656 nm, the diffraction-limited optical resolution (i.e., Airy disk width) ranged from 260 to 420 nm, below the minimum length of the nanostructures but well above their maximum width. Accordingly, from Figure 5, there are visual differences in length but all nanostructures appear to have the same width. The two different widths instead exhibit a clear brightness difference between them at this illumination wavelength.

**Figure 6:**
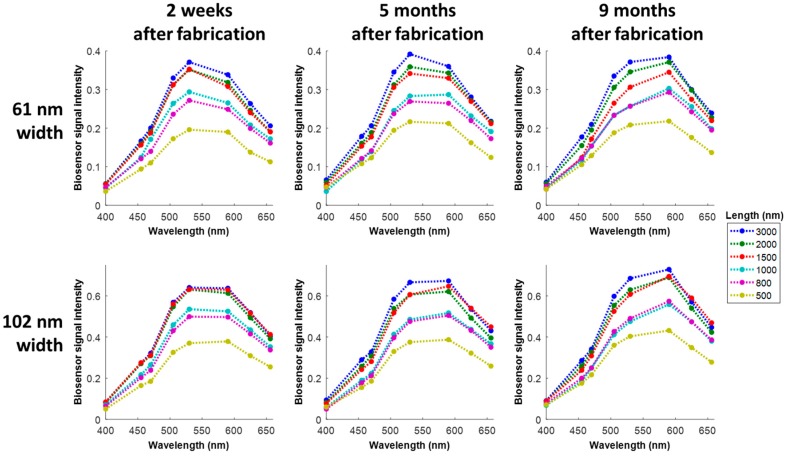
Biosensor spectra measured from the vertical nanostructures at different points in time. Each spectrum consists of only eight measured points; dotted lines are to help guide the eye.

To generate a biosensor spectrum, each LED-illuminated image was first background subtracted, then regions of interest (ROIs) were manually selected in each image enclosing individual objects and one blank region. The final signal intensity (*B*) for an object at a particular wavelength (*λ*) was calculated as:

(1) $$B(\lambda )=\frac{I_{max}(\lambda )}{I_{blank}(\lambda )}-1,$$

where *I_max_* is the mean of the image intensities from the 25 brightest pixels in the object’s ROI and *I_blank_* is the mean image intensity of the blank ROI. Normalizing by the blank intensity accounts for the illumination level at each wavelength. If the object ROI were also blank, then ideally (i.e., with zero noise and spatially uniform illumination) *I_max_*/*I_blank_* would be unity, so subtracting one sets the baseline value of *B* to zero. Figure 6 shows graphs of the biosensor spectra from the vertical nanostructures at three different time points: two weeks after fabrication, then 5 and 9 months after fabrication. In between imaging sessions, the chip was stored in a Petri dish at room temperature on a laboratory bench exposed to room lights. Except for values at 400 nm where the structures have very low reflectivity, intensities clearly increase with structure length and width across the entire spectrum, as expected, since a larger object generally tends to scatter more light. Apparent changes in the spectra over time can be attributed to HSQ/substrate material property changes (e.g., due to hygroscopicity), operator focusing variability across imaging sessions, and any long-term drift in our optical imaging setup. At any given timepoint, the shape of the spectra is markedly consistent across the different nanostructure lengths and widths.

**Figure 7:**
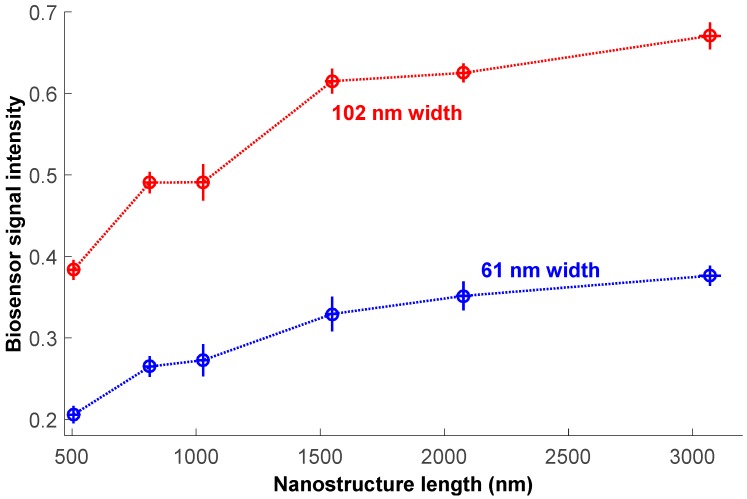
Signal intensity at 530 nm for the six different lengths and two different widths of nanostructures. Horizontal error bars represent one standard deviation of the measured length; vertical error bars represent one standard deviation of the intensity across 9 months of imaging sessions. Dotted lines are to help guide the eye.

The relationship between signal at 530 nm and nanostructure size shown in Figure 7 exemplifies that structure width has a much more profound effect on the signal than length at filovirus size scales: a less than two-fold increase in width roughly doubles the signal intensity, but a six-fold length increase yields less than double the signal. The uncertainty in 530 nm signal intensity, represented as the standard deviation across 9 months of imaging sessions, shows no dependence on structure size. We observed similar results at the other seven wavelengths. Intensity CV over 9 months for each structure at each wavelength ranged between 0.3% and 1.8%.

### 3.3. Nanostructures versus VLPs

**Figure 8:**
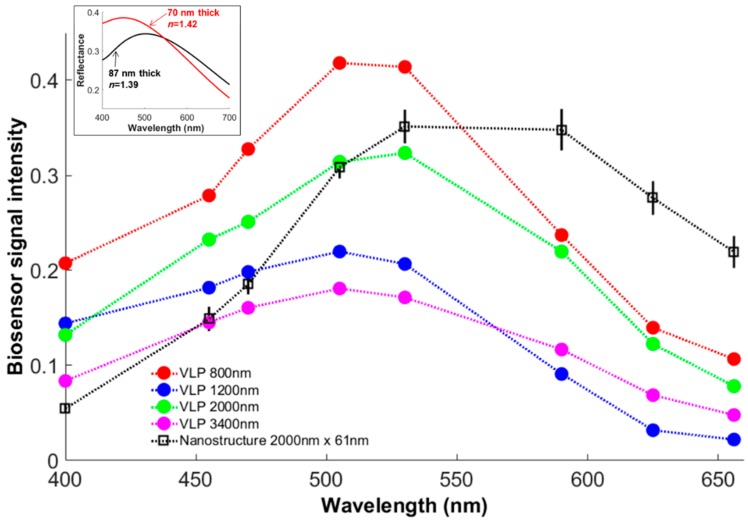
Biosensor spectra from virus-like particles (VLPs) and similarly-sized nanostructure. VLP lengths and nanostructure length and width are indicated in the legend. Vertical error bars on the nanostructure plot represent one standard deviation of the intensity across 9 months of imaging sessions. Dotted lines are to help guide the eye. Inset: Reflectance spectra calculated with Fresnel equations for thin films of different optical thickness on SiO_2_-Si substrate.

Our simple protocol to deposit VLPs on the biosensor substrate was successful but inefficient. An isolated and unobstructed VLP bound to the substrate surface and positively confirmed by fluorescence microscopy was a rare event. Figure 8 shows the representative signals from isolated VLPs of different lengths (measured with fluorescence microscopy) resulting from three deposition sessions. Their overall intensities vary substantially without any clear dependence on length, though all four spectra share a similar shape with a slightly blue-shifted peak compared to the nanostructure spectra. Since we observed spectral shape and peak location to be consistent with nanostructure length and width, the only two remaining variables which could explain the spectral differences between nanostructure and VLP signals are thickness and refractive index, whose product is the well-known physical parameter of optical thickness. This explanation is supported by thin film reflectance calculated with the Fresnel equations [23], which predict a spectral shift with changing optical thickness of a thin film applied to the SiO_2_-Si substrate because of changing levels of constructive and destructive interference (inset of Figure 8). Though the Fresnel-calculated reflectance spectra strictly apply only to films with effectively infinite length and width, they qualitatively corroborate the VLP-nanostructure spectral shift when similar changes to optical thickness are applied in the Fresnel equations. A refractive index difference of 1.42 (virus) − 1.39 (HSQ) = 0.03 is expected, but this difference could be greater since we measured dried VLPs, devoid of genetic material and water present in the viruses characterized by Pang et al. [15]. Instead of falling in the intensity range of our wider (102 nm) nanostructures whose width is close to that of the EBOV virion, the VLP intensities are more similar to those from our narrower (61 nm) nanostructures, likely because dried VLPs shrunk from their native size in aqueous suspension. Therefore, the VLP thickness could also be less than the 87 nm nanostructure thickness.

As a structural approximation of the wild-type EBOV, the VLPs can provide only a general sense of the similarity in signal from nanostructures versus actual filovirus specimens. Even if a VLP produces the identical signal as a filovirus virion, we do not consider the observed spectral shift to be large enough to disqualify the nanostructures as reference materials, which are meant to provide a controlled, stable source of signal for a biosensor resembling a real specimen’s signal but not necessarily identical to it.

### 3.4. Polarization Dependence of Signal

**Figure 9:**
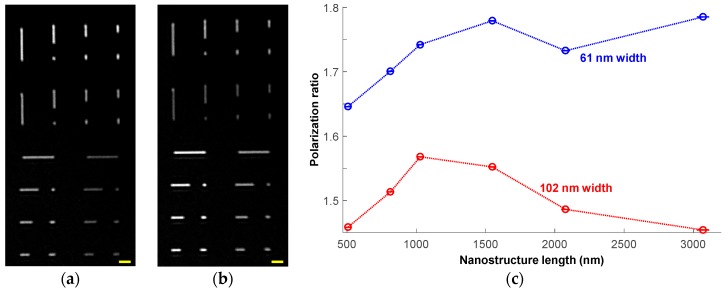
Polarization-induced changes in signal at 530 nm. (**a**) Vertically-polarized image. (**b**) Horizontally-polarized image. Scale bar is 2 μm in both (**a**,**b**). Note alternation of brightness between vertical and horizontal structures between the two images. (**c**) Polarization ratio (vertically-polarized *B*(*λ*) divided by horizontally-polarized *B*(*λ*)) for the six different lengths and two different widths of vertical nanostructures. Horizontal error bars represent one standard deviation of the measured length. Lines are to help guide the eye.

We investigated polarization effects on the signal by capturing images of our nanostructures with the linear polarizer inserted in the light collection path and rotated to image two polarization states: vertical (i.e., aligned parallel with our vertical nanostructures) and horizontal. Figure 9a,b show images at 530 nm from the two orthogonal polarizations, revealing a distinct intensity dependence with polarization state. The structures aligned parallel to the polarization are substantially brighter than their perpendicular counterparts, a relationship which holds for both vertical and horizontal polarizations. Figure 9c plots the polarization ratio (vertically-polarized *B*(*λ*) divided by horizontally-polarized *B*(*λ*)) for the vertical nanostructures at 530 nm. Unlike the original signal, the polarization ratio is larger for the narrower structures and there is no clear monotonic length dependence. Even the shortest structures, 1000 nm and below, whose filamentous structure becomes less visually apparent, exhibit strong polarization sensitivity of 1.45 or greater. Therefore, polarization sensing provides complementary information to the unpolarized signal intensity with respect to the width of filovirus-scale objects, and combining these measurements can enhance identification and sizing of such strongly asymmetric pathogens. In practice, the pathogen’s orientation need not be known a priori; interrogating multiple linear polarization states over any 90° range could determine the object’s polarization ratio and thus the extent of its asymmetric character.

## 4. Discussion and Conclusions

We have demonstrated successful fabrication and utilization of shelf-stable, biohazard-free nanostructures as reference materials particularly suitable for label-free optical biosensing which relies on scattered light. We implemented our nanostructures to resemble a target pathogen with substantial public health significance—the Ebola virus—for detection with a cutting-edge biosensor platform under development. To the best of our knowledge, this is the first experimental report of the relationships between signal intensity and object dimensions for this biosensor-pathogen combination. As long as a thin film of resist can be coated on the biosensing substrate, EBL can create nanostructures on practically any surface relevant to the myriad of emerging optical biosensing techniques, including those based on other physical phenomena like surface plasmon resonance. The sizes and shapes of nanoscale pathogens that can be mimicked are limited only by the EBL system’s spatial resolution, on the order of 10 nm. Once an EBL protocol has been properly refined, which invariably requires multiple trial and error cycles, replicating modest quantities (tens to hundreds) of biosensing chips with customized nanostructures would be tenable. The EBL protocol could also potentially be modified to liberate nanostructures from a substrate surface into an aqueous suspension, thereby adding even greater versatility to their use as biosensing reference materials.

## Figures and Tables

**Table 1:** Dimensions of fabricated nanostructures. Measured value is the mean ± one standard deviation, with the coefficient of variation (CV) shown in parentheses.

Nanostructure Dimension	Measurement Method	Design Value (nm)	Number of Structures Measured	Measured Value (nm)	% Error (Meas. vs. Design)
Length	SEM	500	4	505 ± 19 (3.8%)	1.1%
		800	4	811 ± 11 (1.3%)	1.4%
		1000	4	1026 ± 18 (1.8%)	2.6%
		1500	4	1539 ± 20 (1.3%)	2.6%
		2000	3	2062 ± 24 (1.2%)	3.1%
		3000	4	3084 ± 32 (1.0%)	2.8%
Width	SEM	40	12	61 ± 3 (5.2%)	53%
		80	12	102 ± 5 (5.3%)	27%
Thickness	AFM	80	24	87 ± 5 (5.6%)	8.8%
